# *Solanum lycopersicum*, a Model Plant for the Studies in Developmental Biology, Stress Biology and Food Science

**DOI:** 10.3390/foods11162402

**Published:** 2022-08-10

**Authors:** Wei Liu, Kui Liu, Daoguo Chen, Zhanquan Zhang, Boqiang Li, Mohamed M. El-Mogy, Shiping Tian, Tong Chen

**Affiliations:** 1 Key Laboratory of Plant Resources, Institute of Botany, Chinese Academy of Sciences, Beijing 100093, China; 2 University of Chinese Academy of Sciences, Beijing 100049, China; 3 Vegetable Crops Department, Faculty of Agriculture, Cairo University, Giza 12613, Egypt

**Keywords:** development, food, fruit quality, fungal pathogen, metabolite

## Abstract

Fruits, vegetables and other plant-derived foods contribute important ingredients for human diets, and are thus favored by consumers worldwide. Among these horticultural crops, tomato belongs to the *Solanaceae* family, ranks only secondary to potato (*S. tuberosum* L.) in yields and is widely cultivated for fresh fruit and processed foods owing to its abundant nutritional constituents (including vitamins, dietary fibers, antioxidants and pigments). Aside from its important economic and nutritional values, tomato is also well received as a model species for the studies on many fundamental biological events, including regulations on flowering, shoot apical meristem maintenance, fruit ripening, as well as responses to abiotic and biotic stresses (such as light, salinity, temperature and various pathogens). Moreover, tomato also provides abundant health-promoting secondary metabolites (flavonoids, phenolics, alkaloids, etc.), making it an excellent source and experimental system for investigating nutrient biosynthesis and availability in food science. Here, we summarize some latest results on these aspects, which may provide some references for further investigations on developmental biology, stress signaling and food science.

## 1. Introduction

Tomato (*Solanum lycopersicum*) is one of the best studied cultivated dicotyledonous plants [1]. Its domestication has mainly experienced two stages, represented by *S. pimpinellifolium* and *S. lycopersicum* var. cerasiforme as the ancestor, while persistent breeding of modern cultivars mainly concerns the combinations of various traits to meet with market requirements [1,2]. As one of the common vegetables originated from Central and South America, tomato is favored by numerous consumers for its delicious taste and high nutritional value, and have thus become one of the most economically important crops since the 16th century [1]. According to the report of FAO, the total yield of tomato equals up to 182.05 million tons worldwide and the cultivation area is 5.06 million hectares in 2020; developing countries in Asia contribute more than 1/3 to the total yield [3]. Because of their economic importance, agronomic traits directly affecting ultimate fruit yield, such as branching pattern, inflorescence architecture and fruit development, have been paid additional attention by scientists [4]. Many genes involved in yield-related traits have been identified in tomato, including *SELF PRUNING* (*SP*), *LOCULE NUMBER* (*LC*), *FASCIATED* (*FAS*), *TERMINATING FLOWER* (*TMF*), etc. [4,5].

In addition, tomato has plenty of nutritional traits, making it popular among people all over the world. According to USDA statistics, it is one of the low-calorie vegetables (extremely low fat and zero cholesterol), with only 18 calories per 100 g [6]. Tomato is also an excellent source of antioxidants, dietary fiber, minerals, and vitamins. Taking antioxidants, for example, tomato has a total oxygen radical absorbance capacity at 367 µmol TE/100 g, attributed to the high levels of vitamin A and flavonoid antioxidants, such as α and ß-carotenes, xanthins and lutein. These antioxidants have potent efficacies in scavenging harmful oxygen-free radicals, thus improving people’s night vision and protecting them from aging, tumors and other health-related problems [6]. It should be noted that domestication and genetic breeding of tomato during the past several decades have resulted in decreased genetic diversity and unexpected loss of some important traits, including flavor, aroma and resistance [7,8]. With the continuous change in lifestyle and living standard, people have made efforts to reintroduce the lost traits from wild relatives into commercial cultivars [5,9,10]. Because of tomato’s virtues and the aforementioned situations, tomato has been received as a model species for studies in life science and food science.

## 2. *S. lycopersicum* Is Used for Investigating Inflorescence Branching and Fate Determination of Shoot Apical Meristem

Tomato is a species which undergoes sympodial growth, whose stem cell activity directly determines plant architecture, flowering time, inflorescence structure, fruit size, harvest index and final fruit yield [11,12], composing the developmental basis for molecular design and genetic breeding of tomato cultivars. Therefore, tomato has been widely used as an excellent material for investigating the mechanisms underlying inflorescence branch, flowering and fate determination of shoot apical meristem (SAM) [13,14]. Among the developmental regulators, reactive oxygen species (ROS) produced simultaneously with respiration have important roles in regulating growth, development and responses to unfavorable conditions of plants [15,16]. As the most stable form of ROS, H_2_O_2_ is also accumulated in the SAM of normally grown tomato plants, while the accumulation site coincides with the expression site of TMF, a transcription factor controlling the maturation of SAM, suggesting that ROS may be related to the maturation of SAM [17]. It was found that locally accumulated H_2_O_2_ in SAM drove reversible liquid–liquid phase separation of TMF in tomato [13]. In response to cellular redox status variation, cysteine residues in TMF may form disulfide bonds, which are necessary for connecting multiple TMF molecules and further increasing intrinsically disordered regions to trigger phase separation. Such phase separation enables TMF to suppress the expression of *ANANTHA*, a floral identity gene [13]. This characteristic reversible biomolecular condensate may provide a suitable microenvironment for rapid transcriptional programming via redox-regulated phase separation. These results well correlate liquid–liquid phase separation of proteins, redox signaling and cell fate determination of SAM, thereby assuring tomato as an excellent example for dissecting complex agronomic traits encompassing developmental robustness and stress responses.

Functional redundancy of gene families is always caused by gene duplications and gene family expansion, resulting in so-termed phenotypic robustness [18]. However, how such phenotypic robustness is maintained in the specific cellular contexts is still seldom studied in plants. The fate transition of SAM is a well-regulated programmed developmental process which is required to be initiated and completed at the right time and right place [17]. Untimely transition may lead to failure to adapt to environmental changes or crop yield loss [11]. The precise control of tomato stem cell fate transition is determined by ALOG transcription factors. TMF, the first ALOG transcription factor identified from tomato, functions to maintain meristem at a vegetative state [19]. During gene duplication and gene family expansion, some mutations were introduced in the *cis*-regulatory sequences and coding sequences of ALOG genes, maintaining the activity of the four *TFAM* genes (TMF family member) (*TFAM1/2/3/11*) in SAM [19]. TFAM1/2/3/11 proteins all contain typical intrinsically disordered regions (IDRs) and undergo liquid–liquid phase separation [14]. Interestingly, although the ALOG domain is conserved, many mutations occur to the N-terminal and C-terminal IDRs, leading to differences in protein phase separation and transcriptional regulation [14]. Following site-directed mutation, IDR replacement and transcriptional activity analysis, it was confirmed that the realization of the robustness of tomato cell fate transition depends on the ALOG transcription factors to form heterogeneous biomolecular condensates through protein phase separation, thereby precisely regulating the spatio-temporal expression of *ANATHA* (*AN*) [14]. Consequently, tomatoes can blossom and bear fruit in the right place at the right time. This work represents another excellent example for investigating fate determination of SAM using tomato as a model system.

## 3. *S. lycopersicum* Is a Model Material for Studies on Fruit Ripening Regulation

**Figure 1:**
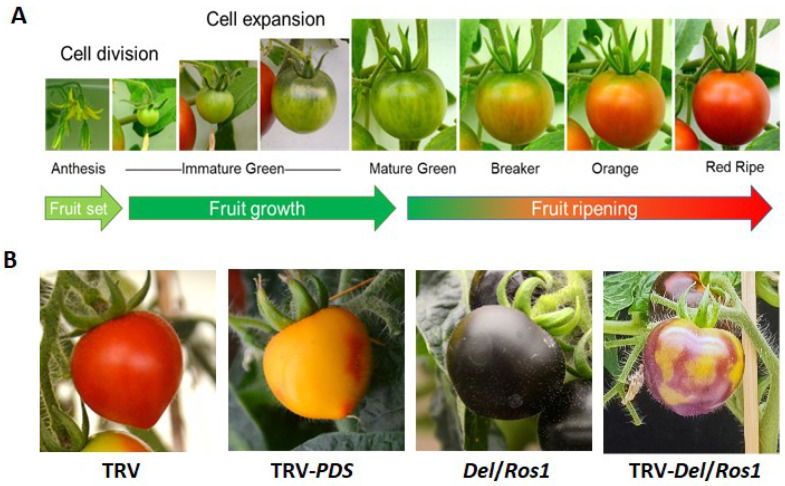
Tomato fruit development and virus-induced gene silencing. (**A**) Tomato fruit development. (**B**) Phenotypes for VIGS-PDS fruit (*S. lycopersicum* cv MicroTom and *S. lycopersicum Del*/*Ros1* cv MicroTom).

Some fruits, particularly climacteric fruit, are harvested at lower maturity and serve as foods for consumers at appropriate maturity by postharvest processing at harvest, storage, delivery and retailing [20]. Therefore, it is necessary to manipulate the ripening processing and maintain the fruit quality to provide delicious foods at the right time for consumers [21]. Owing to a relatively small genome (~900 Mb), a relatively short life cycle, well dissected genetic background, extensive germplasm collection and mature experimental procedures for genetic transformation, tomato has become a model plant for the studies on developmental biology of climacteric fruit [20,22]. Tomato fruit development can be categorized into two major developmental processes: early fruit development and fruit ripening, whereas the morphological structure and size of fruit are mainly determined at the early fruit development stage [23]. The early development of tomato fruit can be further divided into three stages: (1) ovary development and fruit setting; (2) rapid division of the cells and significant increase in the number of cells; (3) trigger of growth and development of tomato fruit. Tomato fruit ripening has obvious stage characteristics, by which the ripening process is always categorized as immature green (IMG), mature green (MG), breaker (Br), orange (Or) and red ripe (RR) according to the changes in fruit color and firmness [21] (Figure 1A). Importantly, fruit ripening is accompanied by the formation of many fruit quality-related traits, such as firmness, color, aroma, vitamins and others [22].

Among the methods for analyzing ripening-related traits, virus-induced gene silencing (VIGS) has been proven as an efficient technique for rapid examination of gene functions in plants, particularly for fruit color and other obviously visible phenotypes [24]. In terms of tomato fruit, VIGS offers a rapid alternative for knocking down the expression of a specific gene without tedious genetic transformation through tissue culture [25]. The TRV-based VIGS system can be efficiently introduced by infiltration into detached tomato fruit by either vacuum infiltration or by injection into the fruit attached to the host plant via carpopodium or stem [26]. As definite examples, VIGS-*SlPDS*, *SlEIN2*, *SlCTR1* and *SlEILs* (the genes related to lycopene biosynthesis and ethylene signaling) all resulted in similar ununiform color phenotypes as observed later by stable transformation with RNAi or CRISPR-Cas9 constructs [26], thereby significantly shortening the time required for preliminary identification of gene functions. Similarly, VIGS technique has also been applied to develop a visual reporter system in some specific transgenic tomato lines based on anthocyanin accumulation, such as *Del*/*Ros1* [27]. The Del/Ros1 transgenic line expresses *Delila* and *Rosea1* from the ornamental flower snapdragon (*Antirrhinum majus* L.) under the control of the fruit-specific E8 promoter, showing abundant anthocyanin and characteristic purple fruit. VIGS-*Del*/*Ros1* impairs the original purple fruit phenotype of *Del*/*Ros1* line (Figure 1B), whereas simultaneous VIGS of *Del*/*Ros1* and a certain gene of interest may provide a directly visible control for gene expression analysis and precise sampling of different sectors. This may facilitate the functional dissection for the genes whose knock-down or knockout manipulation may not lead to obviously direct-visible variations.

In terms of the mutants, omics approaches have revealed more insights into regulatory machinery upstream of ethylene and ripening-related signaling systems. The transcription factor MADS-RIN (RIPENING INHIBITOR) is a ripening-related key regulator that has been extensively investigated during the past several decades [28,29]. *Rin* mutant has defects in fruit softening and does not exhibit the characteristic respiratory rise and ethylene peak [30]. However, it has been suggested that *rin* mutation resulted in the fusion of truncated *RIN* to *Macrocalyx* (*RIN*-*MC*), which produced a gain-of-function chimeric protein transcriptionally repressing fruit ripening [28,31]. Coincidently, knockout of SBP-CNR and NAC-NOR transcription factors by CRISPR/Cas9 genome editing only led to postponed or partially non-ripening phenotypes, which were significantly different from the phenotypes observed in the spontaneous mutants [32]. No alteration was detected in the *SPL-CNR* DNA sequence; however, its promoter accumulated hypermethylated modification, giving rise to inhibition on the transcription of the *SPL-CNR* gene and the *Cnr* mutant phenotype [33]. Similarly, the non-ripening (*nor*) mutant of tomato also harbors a truncated protein (NOR186) composed of 186 amino acids due to two adenine deletion in *NAC*-*NOR*, leading to a gain-of-function mutant in which the transcriptional activation domain was interrupted but the DNA-binding domain was retained (US Patent, No. US 6762347B1). NAC-NOR overexpression in the *nor* mutant did not fully complement the defects in ripening-related phenotypes, while the truncated NOR186 competed with the wild-type NOR for binding to the promoter regions of 1-aminocyclopropane-1-carboxylic acid synthase2 (*SlACS2*), pectate lyase (*SlPL*) and geranylgeranyl diphosphate synthase2 (*SlGgpps2*) [34]. Collectively, previous models depicting RIN, NOR and other regulators as indispensable for fruit ripening should be reconsidered [20,29,31,34], while the integration of state-of-art molecular biological techniques and data mining may provide solutions to these unsolved questions in tomato fruit.

## 4. *S. lycopersicum* Is a Classic Model Species for Studies on Responses to Abiotic Stress

Under significant global climate changes, plants are persistently confronted with various unfavorable environmental conditions (such as high light, low/high temperature, drought, salinity, etc.) during their life cycles, which oblige them to successfully evolve sophisticated signaling network to cope with such harsh conditions [35,36]. Tomato has also been well utilized to investigate the responsive machinery to environmental stimuli [36].

Responses to low light and high light. Light is above all the most important signal for plants, and is indispensable for photosynthetic biomass production and resistance to pathogens [37]. A recent study demonstrated that tomato was more susceptible to *Pst* DC3000 under low light, which was attributed to the decreased apoplastic glucose. Further studies revealed that the regulator of G protein signaling 1 (RGS1)/heterotrimeric G protein was activated to negatively regulate the defense responses of tomato; however, this regulation was independent of SA or JA signaling [38]. These results correlate the signaling of plants in response to abiotic and biotic stress, which may provide certain clues for developing future strategies for efficient production of protected vegetables. In contrast, high light intensity may lead to photooxidative stress, bursts of reactive oxygen species (ROS) and subsequent responses [39]. In a lipidomics study to identify lipophilic molecules that enhance tolerance after exposure to combined high-temperature and high-light stress, alpha-tocopherol and plastoquinone/plastoquinol were the most significantly upregulated, which may contribute to the protection of photosystem II (PSII) over photodamage under environmental stress [40]. Interestingly, certain light intensity and quality may induce the accumulation of specific metabolites [41,42], among which steroidal glycoalkaloids (SGAs) and anthocyanins are two groups. SGAs are specialized secondary metabolites mainly produced in Solanaceous species [8], which are toxic for humans and contribute to defense responses. It was found that SlHY5 (ELONGATED HYPOCOTYL 5) and SlPIF3 (PHYTOCHROME INTERACTING FACTOR3) directly bound to respective elements in the promoter regions and transcriptionally activated the key genes involved in SGA biosynthesis, namely GAME1, GAME4 and GAME17, thus modulating the transcript abundance of these enzymes [42]. These results provide a potential genetic basis for genetic manipulation of SGA level in Solanaceous crops. As major water-soluble pigments, anthocyanins have important roles in protecting plant tissues from being damaged by high light, UV radiation, and other unfavorable environmental factors [27]. High light induced a nonuniform anthocyanin pigmentation pattern in the *Pro35S:BrTT8* tomato fruit. This was attributed to the induction of SlAN2 expression at high level in the upper part of fruit after exposure to high light, resulting in efficient local assembly of MBW complex (a protein complex composed of MYB, bHLH and WDR protein) with BrTT8 and SlAN11 [41]. In contrast, the low light treatment failed to activate the genes involved in anthocyanin biosynthesis, while the genes involved in chlorophyll biosynthesis were also markedly suppressed [41], further assuring the feasibility for genetical manipulation of secondary metabolites under artificial illumination environment.

Responses to salinity. Most tomato cultivars are sensitive to salinity during their life, particularly at the seed germination and early seedling stages, while natural variation in salinity tolerance has been detected in wild relatives, including *S. pimpinellifolium*, *S. pennellii*, *S. habrochaites* and *S. lycopersicoides* [43]. A couple of famous genes are successively identified in tomato to alleviate the toxicity of excessive Na^+^ [44,45,46,47,48,49]. The plasma membrane Na^+^/H^+^ antiporter SlSOS1 is essential for salinity tolerance by promoting the transport of Na^+^ from underground parts to aboveground parts [44], while the overexpression of SlNHX2, a K^+^/H^+^ antiporter, led to an enhanced K^+^ sequestration capacity and higher salinity tolerance [45]. In contrast, the *SlSOS2* (a gene encoding a protein kinase) overexpressing plants displayed higher salinity tolerance by promoting Na^+^ export and compartmentation [46]. Attempts have been made to enhance salinity tolerance in tomatoes by genetic engineering on these genes that encode Na^+^/H^+^ antiporters SlSOS1 and SlNHX2, as well as regulatory proteins (including SlSOS2). As a major challenge for plant growth, development and productivity, salt stress often triggers significant transcriptional reprogramming, underpinning the importance of transcription factors in salt stress responses [50]. Transcription factors of diverse families are closely correlated with the responses of tomato to salt stress. *SlAREB1* and *SlAREB2* (the genes encoding ABA-responsive element-binding proteins) were induced by salinity in both roots and leaves. The genes encoding oxidative stress-related proteins, lipid transfer proteins and late embryogenesis abundant proteins were up-regulated in the *SlAREB1*-overexpressing plants, which may be responsible for the higher salt tolerance [47]. SlARS1 (altered response to salt stress 1) was a R1-type MYB transcription factor [48]. The *ars1* mutant displayed defects in stomatal closure in an abscisic acid (ABA)-dependent manner, while the *SlARS1* overexpression line did not show chlorosis but improved water use efficiency, further implying a potential role of ABA signaling in the salt tolerance conferred by *SlARS1*. SlDREB2 encoded a dehydration-responsive element-binding protein, which improved the salt tolerance by elevating K^+^/Na^+^ ratio as well as proline and polyamine contents, while ABA signaling was also involved in the tolerance conferred by SlDREB2 [49]. It should be noted that some key components as mentioned above are involved in the responses to abiotic and biotic stress, further suggesting the necessity of dissecting the crosstalk of these signaling events and exploring key determinants involved [51].

Responses to cold stress. Cold stress is among the major abiotic stress restricting geographical distribution, normal growth and crop yield [52]. Many cold-activated signaling pathways are induced to protect plants from injuries during cold stress. Similar to its counterpart in Arabidopsis, ICE1-CBF-COR module is conserved and also works in cold responses of tomato, as the expression of *SlCBF1* and *SlCBF2* are significantly upregulated after 3 h at 10 °C during cold acclimation in tomato. Moreover, overexpression of *SlICE1* enhanced chilling tolerance by elevating the expression of *SlCBF1*, *SlDRCi7* and *SlP5CS* and the antioxidant capacity of tomato [53]. The transgenic SlICE1 overexpressing lines also accumulated higher levels of pigments and antioxidants, including β-carotene, lycopene and ascorbic acid, as well as certain amino acids and amines, suggesting that the enhanced chilling tolerance may be partly attributed to the improved antioxidant capacity [54]. In addition, cold stress may also affect fruit flavor and other quality traits, as refrigeration at low temperature can delay fruit ripening and prolong the storability of tomato [55,56]. Consumers have begun to complain about the loss of flavor after cold storage during production. An integrative analysis on the transcriptome and flavor metabolome of tomato fruit showed that flavor-related volatiles lost after low temperature storage, while fruit sugar and acid contents did not change. The reduction in the expression of volatile synthesis genes and transcription factor genes related to ripening was associated with the methylation status in the promoter regions [56].

Responses to drought. Water shortage is one of the major threats for sustainable yield output, while increasing aridity has further aggravated the situation [57]. *Solanum lycopersicum* cv. M82 has relatively poor tolerance to abiotic stress, while some of its wild relatives, such as *S. pennellii* LA716, has higher tolerance [58]. Two excellent studies generated a collection of 50 introgression lines (ILs) of *S. pennellii*, which greatly facilitated the dissection of the mechanisms underlying drought tolerance in tomato [9,10]. Along with the recurrent parent *S. lycopersicum* cv. M82, the transcriptional profiling for two drought-tolerant lines identified from the introgression lines showed that the genes involved in transcriptional regulation, signalling, cell wall structure, wax biosynthesis, gluconeogenesis, purine and pyrimidine nucleotide biosynthesis, tryptophan degradation, starch degradation, methionine biosynthesis and superoxide radical scavenging were specifically affected by drought stress [59]. Using the near-isogenic lines (NILs) carrying the resistance genes *Ol-1*, *Ol-2* and *Ol-4* to *Oidium neolycopersici*, Sunarti et al. revealed that the growth reduction caused by drought stress was not further aggravated upon powdery mildew (PM) infection, while more severe drought stress resulted in less biomass of PM. Upon drought stress, the NILs harboring *Ol-2*, and *Ol-4* displayed normal resistance and cell death phenotype, while the NIL carrying *Ol-1* showed mildly attenuated resistance [60]. Interestingly, many genes related to hormonal signaling and wound responses, such as *SlNCED*, *SlPR1*, *SlACS2* and *SlLIN6*, were significantly induced, further implying a complex cross-talk of abscisic acid (ABA), salicylic acid (SA), jasmonic acid (JA), ethylene (ET) and wound signaling during combined stress conditions.

## 5. *S. lycopersicum* Is a Classic Model Species for Studies on Responses to Biotic Stress

**Figure 2:**
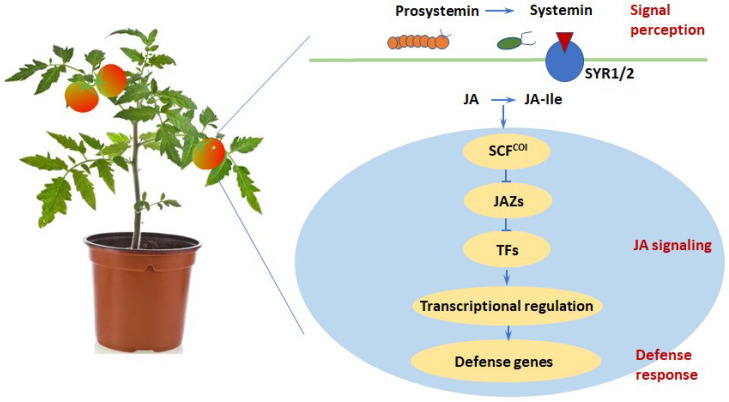
Tomato as a model experimental system for investigating host–pathogen interaction. Upon insect attack or wounding, systemin is processed from prosystemin following proteolytic cleavages and further binds to its putative receptor STR1/2, thus triggering the octadecanoid pathway for JA synthesis. SCF^COI1^ receptor perceives JA, further triggers JAZ repressor degradation and thus relieve the transcription factors to induce the expression of JA-responsive genes for defense.

Tomato has always been a model plant for studying the defense responses of plants to pathogenic microorganisms and insects [61,62], which may provide important reference for the practices in maintaining fruit resistance and reducing food loss. The first *Pto* gene (a resistance gene encoding a serine/threonine kinase) for verifying the “gene to gene” theory was cloned from tomato [63]. Until now, many important resistance-related genes have been cloned from tomato, which are involved in the resistance to fungi, nematodes, aphids, bacteria and viruses [64,65,66,67] (as summarized in Table 1). From injury stimulation to hormonal signaling and resistant gene expression, tomato has been extensively used to explore the jasmonate (JA)-regulated immunity of plants in response to insect attack and other mechanical damages. JA is a phytol-oxylipin hormone modulating growth and immunity of plants by triggering transcriptional programming, in which the basic helix-loop-helix transcription factor MYC2 and MED25 (a subunit in the Mediator complex) play a central role. In the presence of the JA ligand jasmonoyl-isoleucine (JA-Ile), JASMONATE-ZIM DOMAIN (JAZ) proteins form a co-receptor complex with CORONATINE-INSENSITIVE 1 (SCF^COI1^) in a JA-Ile dependent manner, while the SCF^COI1^-dependent degradation of JAZs leads to the release of MYC2; MYC2 thereby activates JA-mediated transcriptional reprogramming of downstream genes [68]. Most of the MYC-targeted genes were transcription factors (MYC2-traget transcription factors, MTFs) and proteins related to early JA responses. MYC2 and the MTF JA2-like preferentially regulate wound-responsive genes, while MYC2 and ETHYLENE RESPONSIVE FACTOR.C3 (also an MTF) preferentially regulate pathogen responsive genes [64], suggesting a differential activation of wound-responsive and pathogen-responsive transcriptional modules encompassing MYC2. The transcriptional activation of JA-responsive genes also depends on MED25, a subunit in the Mediator transcriptional activation complex [68]. Moreover, MYC2 also regulate the termination of JA signaling by regulating several MYC2-targeted bHLH (MTB) TFs, which exert antagonistic effects on the MYC2-MED25 transcriptional activation complex by competing with MYC2 for its targets [66]. In addition to MYC2 and RNA polymerase II, MED25 also interacts with other regulators involved in MYC2-dependent transcription, including hormone receptor activation, transcriptional termination and epigenetic regulation (Figure 2) [68], thereby affecting downstream responses of tomato to pathogen and insect invasion.

Tomato is significantly affected by an array of pathogens (including bacteria, fungi, oomycetes, viruses, and nematodes) during cultivation and at postharvest stage [35]. Rapid advances in integrative omics techniques and other novel techniques have facilitated a comprehensive elucidation of the molecular machinery underlying the interaction between tomato and certain pathogens [8,62]. Importantly, tomato fruit–pathogen interaction is an excellent system for investigating the transition from resistance to susceptibility in plants [35,87,88]. Transcriptomic, proteomic and metabolomic techniques have been employed to document the global responses of tomato to infections [8,87,88]. Generally, fruit at low maturity are more resistant to pathogens, but gradually become susceptible when the ripening program is triggered [88]. Accompanied with substantial biochemical and physiological variations, fruit ripening is coordinated by complex signaling events at transcriptional, post-transcriptional and epigenetic levels [20,89]. As revealed by integrative transcriptomic and metabolomic analysis on 580 introgression lines, *S. pennellii* introgressions resulted in the variations for a bulk of transcripts and metabolites in ripening-related processes [8]. α-tomatine, a major glycoalkaloid in tomato, was transformed into esculeosides and lycoperosides during fruit development, which may be important for reducing the bitter taste and the defense responses of fruit. Meanwhile, specific subsets of susceptibility- and resistance-related genes also showed significant variations, while transient knock-down of ACO5, ACD2 and 4CL-like genes resulted in bigger necrotic lesions following *Botrytis cinerea* inoculation, suggesting that these genes may be crucial for the resistance to fungal pathogens in tomato fruit at higher maturity [8]. Blanco-Ulate et al. performed an RNAseq analysis on tomato fruit following *Botrytis cinerea* infection, aiming at examining the gene expression profiles for the signaling of ethylene (ET), abscisic acid (ABA), salicylic acid (SA) and JA [87]. Notably, in a study using different pathogens to infect tomato fruit at varied maturity, besides the common factors (chitinases, PR proteins, WRKY transcription factors, and reactive oxygen species) responding to pathogen infection, susceptible ripe fruit also displayed strong immune responses [88]. As revealed by transcriptional profiling and hormone quantitation, susceptible tomato genotypes failed to maintain the cellular redox homeostasis, whereas endogenous JA production and signaling were normally activated as observed in resistant fruit. These results collectively suggest that the interaction between tomato plants (fruit) and pathogens may provide important reference for modulating quality traits and alleviating fungal diseases.

## 6. *S. lycopersicum* Is an Excellent Material for Studies on Nutritional Metabolite Synthesis and Other Topics in Food Science

As an important component in human diets, tomato has also been an excellent experimental material for food science owing to abundant beneficial nutrients and antioxidants [1,5]. Besides sugars, organic acids, amino acids and other primary metabolites, tomato is also abundant in pigments, flavonoids, volatiles and other secondary metabolites [90,91]. Moreover, tomato also has some specific secondary metabolites that accumulate at millimolar levels, which display antimicrobial and antinutritional activity, such as α-tomatine [92].

Tomato fruit and its processed products always contain high levels of lycopene and β-carotene, thus having been regarded as important sources of lycopene and provitamin A in human diet [7]. Owing to the presence of health-promoting phytochemicals, such as tocopherols and flavonoids in tomato, tomato and its processed products are catalogued as functional foods [6]. Tomato cultivars and transgenic lines have been generated with higher levels of lycopene, β-carotene zeaxanthin and lutein after domestication and genetic breeding [93]. Anthocyanins are also natural pigments ubiquitously distributed in plants [94,95,96]. Butelli et al. generated a characteristic purple tomato line accumulating anthocyanin at high level by ectopic expression of two transcription factors (Del and Rosea1) from *A. majus*. Importantly, these transgenic fruits also significantly prolonged the life span of cancer-susceptible mice, implying additional health-promoting effects and potential benefits of application [97]. Similar anthocyanin-enriched fruits have also been reported for *S. lycopersicum* lines carrying *Aft* gene, *atv* gene and both genes (*Aft* × *atv* plants) as well as *S. lycopersicum* cv. MicroTom overexpressing *AtMYB75* and *SlMYB75* [94,95,96,98,99]. As reported, the *Pro35S:BrTT8* tomato fruit accumulated abundant anthocyanins in an uneven pattern under natural high light, but remained anthocyaninless under low light conditions [41]. Notably, the endogenous *SlTT8* was significantly upregulated in the *Pro35S:BrTT8* transgenic line, implying that *SlTT8* may be regulated by *BrTT8* and other potential regulators, rather than a director target of high light signaling [41], which deserves further clarification. Nevertheless, all these cultivars may serve as excellent materials for the study on anthocyanin pigmentation patterns in fruits exposed to light, cold and other environmental factors, thereby substantially facilitating metabolic engineering on tomato cultivars.

As narrated in a GWAS mapping of quality traits for tomato fruit, robust phenotyping and metabolite profiling technologies have enabled high-throughput quantitation of many growth parameters and variants [4,5]. Meanwhile, the results also demonstrated that genetic breeding globally affected fruit metabolite content as selection of alleles for fruit size and color significantly altered metabolite profiles [7]. In a recent study, Szymanski et al. performed transcriptional, metabolomic and phenotypic profiling in combination with QTL analysis on tomato fruit. The integrated transcriptomic and metabolomic analysis identified a bulk of genomic loci associated with specific transcripts and metabolites. These variations were associated with key constituents in *Solanum* glycoalkaloid biosynthesis, as well as transcripts and metabolites involved in responses to pathogens, thereby providing important data for correlating transcriptional and metabolic reprogramming with pathogen resistance in tomato fruit [8]. Li et al. constructed a global map of for major metabolic variations throughout the growth cycle by integrating spatio-temporal metabolomic and transcriptomic data, thus providing resources for investigating metabolic processes in tomato [90]. These results are valuable for dissecting regulatory mechanisms and further metabolic engineering for flavonoids, polyphenols, steroidal glycoalkaloids, phenylpropanoids and other compounds. These compounds have diversified functions in many important biological processes in plants, including pigmentation of fruits and vegetables, cell wall biofortification, defense responses to pathogen invasion, shields over high-light, drought, chilling and salinity, as well as precursors for aromatic volatiles [100]. Moreover, these compounds comprise an indispensable component of our daily diets, as revealed by numerous benefits of fruits and vegetables in terms of preventing cardiovascular diseases, vitamin deficiency, obesity and other chronic diseases [101,102]. Metabolic engineering has brought about many surprises and may fundamentally change our lifestyle in the near future. Given the newly reported existence of a ‘duplicate’ pathway for steroidal glycoalkaloid (SGA) biosynthesis in tomato, an excellent study reported exciting results for promoting the accumulation of provitamin D_3_ in tomato by gene editing for 7-dehydrocholesterol reductase gene (*Sl7-DR2*) [103]. As compared with the wild type of fruit, the *Sl7-DR2*-knockout homozygous lines accumulated 7-dehydrocholesterol at higher levels in both leaves and mature green fruit, which is known as provitamin D_3_, thereby modifying phytosterol biosynthesis pathway in tomato fruit [103]. According to estimates, the provitamin D_3_ in tomato may be comparable to that in two eggs of average size, a dose recommended for vitamin D by FDA [104]. These results may provide biofortified foods conveniently and efficiently, thus making it possible to alleviate potential risks of neurocognitive diseases, tumors and even mortality.

## 7. Conclusions

In summary, as an excellent material for developmental biology, stress responses and food science, tomato has substantially expanded our understandings of the unknown mysteries in life science and food science. However, we are still confronted with some refractory situations nowadays, such as insufficient food supply and malnutrition in some poverty areas, as well as obesity, diabetes, cardiovascular diseases and other health-related problems due to major changes of lifestyles. Given the rapid progresses in scientific and technological breakthroughs, we can imagine that further in-depth studies using wild tomato relatives and cultivars may undoubtedly update our knowledge and provide more health-promoting benefits.

We believe that several aspects should be paid additional attention. Firstly, as the global climate changes, extreme weather occurs more frequently and severely than ever, while abiotic and biotic stresses may simultaneously take place during practical tomato production. It is urgent to find out how plants can stimulate their intrinsic defense machineries in response to multiple stresses, so as to provide references for the development of efficient, environmental-friendly and safe strategies to improve broad-spectrum resistance/tolerance in agricultural production. Secondly, CRISPR-Cas9 (clustered regularly interspaced short palindromic repeats/CRISPR-associated proteins 9) technology and other genome editing/modification tools have exhibited great potential to increase food yields and improve food quality, while many selection marker-free transgenic lines have been generated [62,105]. All these genetically edited varieties still require more flexibility and attempts in policy regulations from the authorities. Topically, online sales of genome-editing tomato fruits containing a high level of GABA (ɣ-aminobutyric acid) have started early in Japan this year. This is the first agricultural product in the world to be launched using CRISPR/Cas9 technology [105]. Thirdly, classical breeding techniques should be organically combined with new techniques, thus enriching the modern toolbox used to facilitate the improvements in agronomic traits and nutritional traits [106]. With continuous improvements in people’s requirements for better flavor and longer shelf-life, the way of consuming tomato gradually becomes diversified, and the demand for tomato flavor is increasingly higher no matter whether it is consumed as cooking vegetables or fresh fruit. It is well received by the science community that the research retrieving previously lost quality traits by genetic manipulation and metabolic engineering is beneficial for enriching tomato germplasm resources.

## Figures and Tables

**Table 1:** Representative resistance/tolerance genes from different tomato cultivars.

Gene Name	Pathogen	Disease	Tomato Cultivar	Reference
Fungal disease				
*Asc*	*Alternaria alternata* f. sp. lycopersici	Alternaria stem canker	*S. esculentum*	[69]
*Py*	*Pyrenochaeta lycopersici*	Corky root	*S. hirsutum*	[70]
*Frl*	*Fusarium oxysporum* f. sp. radicis-lycopersici	Crown and root rot	*S. peruvianum*	[71]
*Ph1*, *Ph2*, *Ph3*, *Ph4*, *Ph5*, *Ph6*	*Phytophthora infestans*	Late blight	*S. pimpinellifolium*, *S. habrochaites*	[72]
*Cf* (*Cf-2*, *Cf-5*, *Cf-9*, etc.)	*Cladosporium fulvum*	Leaf mold	*S. peruvianum*	[73]
*Sm*	*Stemphylium lycopersici*, *S. solani*, *S. floridanum*	Gray leaf spot	*S. pimpinellifolium*	[74]
*Lv*	*Leveillula taurica*	Powdery mildew	*S. chilense*	[75]
*Ol-1*, *Ol-3*, *Ol-4*, *Ol-5*, *Ol-6*	*Oidium neolycopersici*		*S. hirsutum*	[76]
*I*, *I2*	*Fusarium oxysporum* f. sp. lycopersici race 1	Wilt	*S. pimpinellifolium*	[77,78]
*I3*, *I7*	*Fusarium oxysporum* f. sp. lycopersici race 2		*S. pennellii*	[77,78]
*Ve1*, *Ve2*	*Verticilliium dahliae*	Verticillium wilt	*S. esculentum*	[79]
Bacterial disease				
*Pto*	*Pseudomonas syringae* pv. tomato	Bacterial speck	*S. pimpinellifolium*	[80]
*Rx4*, *RxLA1589*, *Xv3*, *RXopJ4*	*Xanthomonas* *perforans* race T3	Bacterial spot of tomato	*S. pimpinellifolium* (*Rx4 and RxLA1589*), *Hawaii7981* (*Xv3*), *S. pennellii* (*RXopJ4*)	[81,82]
Viral disease				
*Tm-1*, *Tm-2*, *Tm2^2^*	Tomato mosaic virus (ToMV), tomato chlorotic spot virus (TCSV), groundnut ringspot virus (GRSV), and chrysanthemum stem necrosis virus (CSNV)	Mosaic	*S. hirsutum*, *S. peruvianum*	[83]
*Sw* (*Sw-5a* and *Sw-5b*)	Tomato spotted wilt virus (TSWV)	Spotted wilt	*S. peruvianum*	[84]
*Ty-1*, *Ty-2*, *Ty-3*, *Ty-4*, *Ty-5*, *Ty-6*	Tomato yellow leaf curl virus (TYLCV)	Yellow leaf curl	*S. chilense* (*Ty-1*, *Ty-3*, *Ty-4*, *Ty-6*), *S. habrochaites* (*Ty-2*), *S. peruvianum* (*Ty-5*)	[85]
Nematode disease				
*Mi1*, *Mi3*, *Mi5*, *Mi9*	*Meloidogyne incognita*		*S. peruvianum*	[86]
Aphids				
*Mi1*	*Macrosiphum euphorbiae*		*S. peruvianum*	[86]

Modified and updated from [35].

## Data Availability

Not applicable.

## References

[1] Bai Y., Lindhout P. (2007). Domestication and breeding of tomatoes: What have we gained and what can we gain in the future?. Ann. Bot..

[2] Lin T., Zhu G., Zhang J., Xu X., Yu Q., Zheng Z., Zhang Z., Lun Y., Li S., Wang X. (2014). Genomic analyses provide insights into the history of tomato breeding. Nat. Genet..

[3] (2020). Food and Agriculture Organization of the United Nations. http://www.fao.org/home/en/.

[4] Mata-Nicolás E., Montero-Pau J., Gimeno-Paez E., Garcia-Carpintero V., Ziarsolo P., Menda N., Mueller L.A., Blanca J., Cañizares J., van der Knaap E. (2020). Exploiting the diversity of tomato: The development of a phenotypically and genetically detailed germplasm collection. Hortic. Res..

[5] Ye J., Wang X., Wang W., Yu H., Ai G., Li C., Sun P., Wang X., Li H., Ouyang B. (2021). Genome-wide association study reveals the genetic architecture of 27 agronomic traits in tomato. Plant Physiol..

[6] USDA National Nutrient Database. https://fdc.nal.usda.gov/fdc-app.html#/food-details/170502/nutrients.

[7] Zhu G., Wang S., Huang Z., Zhang S., Liao Q., Zhang C., Lin T., Qin M., Peng M., Yang C. (2018). Rewiring of the Fruit Metabolome in Tomato Breeding. Cell.

[8] Szymański J., Bocobza S., Panda S., Sonawane P., Cárdenas P.D., Lashbrooke J., Kamble A., Shahaf N., Meir S., Bovy A. (2020). Analysis of wild tomato introgression lines elucidates the genetic basis of transcriptome and metabolome variation underlying fruit traits and pathogen response. Nat. Genet..

[9] Eshed Y., Abu-Abied M., Saranga Y., Zamir D. (1992). Lycopersicon esculentum lines containing small overlapping introgressions from *L. pennellii*. Theor. Appl. Genet..

[10] Eshed Y., Zamir D. (1994). A genomic library of Lycopersicon pennellii in *L. esculentum*: A tool for fine mapping of genes. Euphytica.

[11] Park S.J., Eshed Y., Lippman Z.B. (2014). Meristem maturation and inflorescence architecture—Lessons from the Solanaceae. Curr. Opin. Plant Biol..

[12] Thouet J., Quinet M., Ormenese S., Kinet J.-M., Périlleux C. (2008). Revisiting the involvement of SELF-PRUNING in the sympodial growth of tomato. Plant Physiol..

[13] Huang X., Chen S., Li W., Tang L., Zhang Y., Yang N., Zou Y., Zhai X., Xiao N., Liu W. (2021). ROS regulated reversible protein phase separation synchronizes plant flowering. Nat. Chem. Biol..

[14] Huang X., Xiao N., Zou Y., Xie Y., Tang L., Zhang Y., Yu Y., Li Y., Xu C. (2022). Heterotypic transcriptional condensates formed by prion-like paralogous proteins canalize flowering transition in tomato. Genome Biol..

[15] Wang Y., Ji D.C., Chen T., Li B.Q., Zhang Z.Q., Qin G.Z., Tian S.P. (2019). Production, signaling and scavenging mechanisms for reactive oxygen species in fruit-pathogen interactions. Int. J. Mol. Sci..

[16] Zhang Z.Q., Chen Y., Li B.Q., Chen T., Tian S.P. (2020). Reactive oxygen species, a key factor to regulate development and pathogenicity of phytopathogenic fungi. Comput. Struct. Biotechnol. J..

[17] Zeng J., Dong Z., Wu H., Tian Z., Zhao Z. (2017). Redox regulation of plant stem cell fate. EMBO J..

[18] Cusack S.A., Wang P., Lotreck S.G., Moore B.M., Meng F., Conner J.K., Krysan P.J., Lehti-Shiu M.D., Shiu S.-H. (2021). Predictive Models of Genetic Redundancy in Arabidopsis thaliana. Mol. Biol. Evol..

[19] MacAlister C.A., Park S.J., Jiang K., Marcel F., Bendahmane A., Izkovich Y., Eshed Y., Lippman Z.B. (2012). Synchronization of the flowering transition by the tomato TERMINATING FLOWER gene. Nat. Genet..

[20] Chen T., Qin G., Tian S. (2020). Regulatory network of fruit ripening: Current understanding and future challenges. New Phytol..

[21] Giovannoni J., Nguyen C., Ampofo B., Zhong S., Fei Z. (2017). The Epigenome and Transcriptional Dynamics of Fruit Ripening. Annu. Rev. Plant Biol..

[22] Lü P., Yu S., Zhu N., Chen Y.-R., Zhou B., Pan Y., Tzeng D., Fabi J.P., Argyris J., Garcia-Mas J. (2018). Genome encode analyses reveal the basis of convergent evolution of fleshy fruit ripening. Nat. Plants.

[23] Klee H.J., Giovannoni J.J. (2011). Genetics and Control of Tomato Fruit Ripening and Quality Attributes. Annu. Rev. Genet..

[24] Becker A. (2013). Virus-Induced Gene Silencing: Methods and Protocols.

[25] Ji D., Cui X., Qin G., Chen T., Tian S. (2020). SlFERL Interacts with S-Adenosylmethionine Synthetase to Regulate Fruit Ripening. Plant Physiol..

[26] Fu D.-Q., Zhu B.-Z., Zhu H.-L., Jiang W.-B., Luo Y.-B. (2005). Virus-induced gene silencing in tomato fruit. Plant J..

[27] Orzaez D., Medina A., Torre S., Fernandez-Moreno J.P., Luis Rambla J., Fernandez-del-Carmen A., Butelli E., Martin C., Granell A. (2009). A visual reporter system for virus-induced gene silencing in tomato fruit based on anthocyanin accumulation. Plant Physiol..

[28] Vrebalov J., Ruezinsky D., Padmanabhan V., White R., Medrano D., Drake R., Schuch W., Giovannoni J. (2002). A MADS-Box Gene Necessary for Fruit Ripening at the Tomato *Ripening-Inhibitor* (*Rin*) Locus. Science.

[29] Li S., Xu H., Ju Z., Cao D., Zhu H., Fu D., Grierson D., Qin G., Luo Y., Zhu B. (2018). The *RIN-MC* Fusion of MADS-Box Transcription Factors Has Transcriptional Activity and Modulates Expression of Many Ripening Genes. Plant Physiol..

[30] Tigchelaar E.C., Mcglasson W.B., Buescher R.W. (1978). Genetic regulation of tomato fruit ripening. Hortsci. A Publ. Am. Soc. Hortic. Sci..

[31] Ito Y., Nishizawa-Yokoi A., Endo M., Mikami M., Shima Y., Nakamura N., Kotake-Nara E., Kawasaki S., Toki S. (2017). Re-evaluation of the rin mutation and the role of RIN in the induction of tomato ripening. Nat. Plants.

[32] Gao Y., Zhu N., Zhu X., Wu M., Jiang C.-Z., Grierson D., Luo Y., Shen W., Zhong S., Fu D.-Q. (2019). Diversity and redundancy of the ripening regulatory networks revealed by the fruitENCODE and the new CRISPR/Cas9 CNR and NOR mutants. Hortic. Res..

[33] Manning K., Tör M., Poole M., Hong Y., Thompson A.J., King G.J., Giovannoni J.J., Seymour G.B. (2006). A naturally occurring epigenetic mutation in a gene encoding an SBP-box transcription factor inhibits tomato fruit ripening. Nat. Genet..

[34] Gao Y., Wei W., Fan Z., Zhao X., Zhang Y., Jing Y., Zhu B., Zhu H., Shan W., Chen J. (2020). Re-evaluation of the nor mutation and the role of the NAC-NOR transcription factor in tomato fruit ripening. J. Exp. Bot..

[35] Arie T., Takahashi H., Kodama M., Teraoka T. (2007). Tomato as a model plant for plant-pathogen interactions. Plant Biotechnol..

[36] Kissoudis C., Sunarti S., van de Wiel C., Visser R.G., Van Der Linden C.G., Bai Y. (2016). Responses to combined abiotic and biotic stress in tomato are governed by stress intensity and resistance mechanism. J. Exp. Bot..

[37] Genoud T., Buchala A.J., Chua N.-H., Métraux J.-P. (2002). Phytochrome signalling modulates the SA-perceptive pathway in *Arabidopsis*. Plant J..

[38] Wang J., Wang A., Luo Q., Hu Z., Ma Q., Li Y., Lin T., Liang X., Yu J., Foyer C.H. (2022). Glucose sensing by regulator of G protein signaling 1 (RGS1) plays a crucial role in coordinating defense in response to environmental variation in tomato. New Phytol..

[39] Steelheart C., Alegre M.L., Baldet P., Rothan C., Bres C., Just D., Okabe Y., Ezura H., Ganganelli I.M., Grozeff G.E.G. (2022). High light stress induces H_2_O_2_ production and accelerates fruit ripening in tomato. Plant Sci..

[40] Spicher L., Almeida J., Gutbrod K., Pipitone R., Dörmann P., Glauser G., Rossi M., Kessler F. (2017). Essential role for phytol kinase and tocopherol in tolerance to combined light and temperature stress in tomato. J. Exp. Bot..

[41] Zhang Y., Li Y., Li W., Hu Z., Yu X., Tu Y., Zhang M., Huang J., Chen G. (2019). Metabolic and molecular analysis of nonuniform anthocyanin pigmentation in tomato fruit under high light. Hortic. Res..

[42] Wang C.-C., Meng L.-H., Gao Y., Grierson D., Fu D.-Q. (2018). Manipulation of Light Signal Transduction Factors as a Means of Modifying Steroidal Glycoalkaloids Accumulation in Tomato Leaves. Front. Plant Sci..

[43] Sholi N.J. (2012). Effect of Salt Stress on Seed Germination, Plant Growth, Photosynthesis and Ion Accumulation of four Tomato Cultivars. Am. J. Plant Physiol..

[44] Olias R., Eljakaoui Z., Li J., De Morales P.A., Marin-Manzano M.C., Pardo J.M., Belver A. (2009). The plasma membrane Na^+^/H^+^ antiporter SOS1 is essential for salt tolerance in tomato and affects the partitioning of Na^+^ between plant organs. Plant Cell Environ..

[45] Huertas R., Rubio L., Cagnac O., Garcia-Sanchez M.J., Alche Jde D., Venema K., Fernandez J.A., Rodriguez-Rosales M.P. (2013). The K^+^/H^+^ antiporter LeNHX2 increases salt tolerance by improving K^+^ homeostasis in transgenic tomato. Plant Cell Environ..

[46] Belver A., Olias R., Huertas R., Rodriguez-Rosales M.P. (2012). Involvement of SlSOS2 in tomato salt tolerance. Bioengineered.

[47] Orellana S., Yañez M., Espinoza A., Verdugo I., González E., Ruiz-Lara S., Casaretto J.A. (2010). The transcription factor SlAREB1 confers drought, salt stress tolerance and regulates biotic and abiotic stress-related genes in tomato. Plant Cell Environ..

[48] Campos J.F., Cara B., Perez-Martin F., Pineda B., Egea I., Flores F.B., Fernandez-Garcia N., Capel J., Moreno V., Angosto T. (2016). The tomato mutant ars1 (altered response to salt stress 1) identifies an R1-type MYB transcription factor involved in stomatal closure under salt acclimation. Plant Biotechnol. J..

[49] Hichri I., Muhovski Y., Clippe A., Žižková E., Dobrev P.I., Motyka V., Lutts S. (2016). SlDREB2, a tomato dehydration-responsive element-binding 2 transcription factor, mediates salt stress tolerance in tomato and Arabidopsis. Plant Cell Environ..

[50] Deinlein U., Stephan A.B., Horie T., Luo W., Xu G., Schroeder J.I. (2014). Plant salt-tolerance mechanisms. Trends Plant Sci..

[51] Devkar V., Thirumalaikumar V.P., Xue G., Vallarino J.G., Turečková V., Strnad M., Fernie A.R., Hoefgen R., Mueller-Roeber B., Balazadeh S. (2020). Multifaceted regulatory function of tomato SlTAF1 in the response to salinity stress. New Phytol..

[52] Miura K., Furumoto T. (2013). Cold Signaling and Cold Response in Plants. Int. J. Mol. Sci..

[53] Miura K., Shiba H., Ohta M., Kang S.W., Sato A., Yuasa T., Iwaya-Inoue M., Kamada H., Ezura H. (2012). SlICE1 encoding a MYC-type transcription factor controls cold tolerance in tomato, Solanum lycopersicum. Plant Biotechnol..

[54] Miura K., Sato A., Shiba H., Kang S.W., Kamada H., Ezura H. (2012). Accumulation of antioxidants and antioxidant activity in tomato, Solanum lycopersicum, are enhanced by the transcription factor SlICE1. Plant Biotechnol..

[55] Renard C.M., Ginies C., Gouble B., Bureau S., Causse M. (2013). Home conservation strategies for tomato (*Solanum lycopersicum*): Storage temperature vs. duration—Is there a compromise for better aroma preservation?. Food Chem..

[56] Zhang B., Tieman D.M., Jiao C., Xu Y., Chen K., Fei Z., Giovannoni J.J., Klee H.J. (2016). Chilling-induced tomato flavor loss is associated with altered volatile synthesis and transient changes in DNA methylation. Proc. Natl. Acad. Sci. USA.

[57] Passioura J. (2007). The drought environment: Physical, biological and agricultural perspectives. J. Exp. Bot..

[58] Rick C.M., Srb A.M. (1973). Potential Genetic Resources in Tomato Species: Clues from Observations in Native Habitats. Handbook of Genetics.

[59] Gong P., Zhang J., Li H., Yang C., Zhang C., Zhang X., Khurram Z., Zhang Y., Wang T., Fei Z. (2010). Transcriptional profiles of drought-responsive genes in modulating transcription signal transduction, and biochemical pathways in tomato. J. Exp. Bot..

[60] Sunarti S., Kissoudis C., Van Der Hoek Y., Van Der Schoot H., Visser R.G.F., Van Der Linden C.G., Van De Wiel C., Bai Y. (2022). Drought Stress Interacts with Powdery Mildew Infection in Tomato. Front. Plant Sci..

[61] Campos M.D., Félix M.D.R., Patanita M., Materatski P., Varanda C. (2021). High throughput sequencing unravels tomato-pathogen interactions towards a sustainable plant breeding. Hortic. Res..

[62] Chen T., Ji D., Zhang Z., Li B., Qin G., Tian S. (2021). Advances and Strategies for Controlling the Quality and Safety of Postharvest Fruit. Engineering.

[63] Ronald P.C., Salmeron J.M., Carland F.M., Staskawicz B.J. (1992). The cloned avirulence gene avrPto induces disease resistance in tomato cultivars containing the Pto resistance gene. J. Bacteriol..

[64] Du M., Zhao J., Tzeng D.T., Liu Y., Deng L., Yang T., Zhai Q., Wu F., Huang Z., Zhou M. (2017). MYC2 Orchestrates a Hierarchical Transcriptional Cascade That Regulates Jasmonate-Mediated Plant Immunity in Tomato. Plant Cell.

[65] Wang W., Cai J., Wang P., Tian S., Qin G. (2017). Post-transcriptional regulation of fruit ripening and disease resistance in tomato by the vacuolar protease SlVPE3. Genome Biol..

[66] Liu Y., Du M., Deng L., Shen J., Fang M., Chen Q., Lu Y., Wang Q., Li C., Zhai Q. (2019). MYC2 Regulates the Termination of Jasmonate Signaling via an Autoregulatory Negative Feedback Loop. Plant Cell.

[67] Cai J., Chen T., Wang Y., Qin G., Tian S. (2020). SlREM1 Triggers Cell Death by Activating an Oxidative Burst and Other Regulators. Plant Physiol..

[68] Zhai Q., Deng L., Li C. (2020). Mediator subunit MED25: At the nexus of jasmonate signaling. Curr. Opin. Plant Biol..

[69] Brandwagt B.F., Kneppers T.J.A., Nijkamp H.J.H., Hille J. (2002). Overexpression of the tomato Asc-1 gene mediates high insensitivity to AAL toxins and Fumonisin B 1 in tomato hairy roots and confers resistance to *Alternaria alternata* f. sp. lycopersici in Nicotiana umbratica plants. Mol. Plant Microbe Interact..

[70] Doganlar S., Dodson J., Gabor B., Beck-Bunn T., Crossman C., Tanksley S.D. (1998). Molecular mapping of the py-1 gene for resistance to corky root rot (*Pyrenochaeta lycopersici*) in tomato. Theor. Appl. Genet..

[71] Berry S.Z., Oakes G.L. (1987). Inheritance of resistance to Fusarium crown and root rot in tomato. HortScience.

[72] Chaudhary R., Atamian H.S. (2017). Resistance-gene-mediated defense responses against biotic stresses in the crop model plant tomato. J. Plant Pathol. Microbiol..

[73] Hammondkosack K.E., Jones J.D.G. (1994). Incomplete dominance of tomato Cf genes for resistance to *Cladosporium fulvum*. Mol. Plant Microbe Interact..

[74] Yang H., Zhao T., Jiang J., Wang S., Wang A., Li J., Xu X. (2017). Mapping and screening of the tomato *Stemphylium lycopersici* resistance gene, Sm, based on bulked segregant analysis in combination with genome resequencing. BMC Plant Biol..

[75] Chunwongse J., Doganlar S., Crossman C., Jiang J., Tanksley S.D. (1997). High-resolution genetic map of the Lv resistance locus in tomato. Theor. Appl. Genet..

[76] Bai Y., van der Hulst R., Bonnema G., Marcel T.C., Meijer-Dekens F., Niks R.E., Lindhout P. (2005). Tomato defense to Oidium neolycopersici: Dominant Ol genes confer isolate-dependent resistance via a different mechanism than recessive ol-2. Mol. Plant Microbe Interact..

[77] Simons G., Groenendijk J., Wijbrandi J., Reijans M., Groenen J., Diergaarde P., Van der Lee T., Bleeker M., Onstenk J., de Both M. (1998). Dissection of the fusarium I2 gene cluster in tomato reveals six homologs and one active gene copy. Plant Cell.

[78] Catanzariti A.-M., Lim G.T.T., Jones D.A. (2015). The tomato I-3 gene: A novel gene for resistance to Fusarium wilt disease. New Phytol..

[79] Kawchuk L.M., Hachey J., Lynch D.R., Kulcsar F., Rooijen G.V., Waterer D.R., Robertson A., Kokko E., Byers B., Howard R.J. (2001). Tomato Ve disease resistance genes encode cell surface-like receptors. Proc. Natl. Acad. Sci. USA.

[80] Martin G.B. (1994). Analysis of the molecular basis of Pseudomonas syringae pv. tomato resistance in tomato. Euphytica.

[81] Robbins M.D., Darrigues A., Sim S.-C., Masud M.A.T., Francis D.M. (2009). Characterization of Hypersensitive Resistance to Bacterial Spot Race T3 (*Xanthomonas perforans*) from Tomato Accession PI 128216. Phytopathology.

[82] Sharlach M., Dahlbeck D., Liu L., Chiu J., Jiménez-Gómez J.M., Kimura S., Koenig D., Maloof J.N., Sinha N., Minsavage G.V. (2012). Fine genetic mapping of RXopJ4, a bacterial spot disease resistance locus from Solanum pennellii LA716. Theor. Appl. Genet..

[83] Motoyoshi F., Ohmori T., Murata M. (1996). Molecular characterization of heterochromatic regions around the Tm-2 locus in chromosome 9 of tomato. Symp. Soc. Exp. Biol..

[84] de Oliveira A.S., Boiteux L.S., Kormelink R., Resende R.O. (2018). The Sw-5 gene cluster: Tomato breeding and research toward Orthotospovirus disease control. Front. Plant Sci..

[85] Butterbach P., Verlaan M.G., Dullemans A., Lohuis D., Visser R.G.F., Bai Y., Kormelink R. (2014). Tomato yellow leaf curl virus resistance by *Ty-1* involves increased cytosine methylation of viral genomes and is compromised by cucumber mosaic virus infection. Proc. Natl. Acad. Sci. USA.

[86] Vos P., Simons G., Jesse T., Wijbrandi J., Heinen L., Hogers R., Frijters A., Groenendijk J., Diergaarde P., Reijans M. (1998). The tomato Mi-1 gene confers resistance to both root-knot nematodes and potato aphids. Nat. Biotechnol..

[87] Blanco-Ulate B., Vincenti E., Powell A.L., Cantu D. (2013). Tomato transcriptome and mutant analyses suggest a role for plant stress hormones in the interaction between fruit and Botrytis cinerea. Front. Plant Sci..

[88] Silva C.J., Abeele C.V.D., Ortega-Salazar I., Papin V., Adaskaveg J.A., Wang D., Casteel C.L., Seymour G.B., Blanco-Ulate B. (2021). Host susceptibility factors render ripe tomato fruit vulnerable to fungal disease despite active immune responses. J. Exp. Bot..

[89] Zhou L., Gao G., Tang R., Wang W., Wang Y., Tian S., Qin G. (2022). m^6^ A-mediated regulation of crop development and stress responses. Plant Biotechnol. J..

[90] Li Y., Chen Y., Zhou L., You S., Deng H., Chen Y., Alseekh S., Yuan Y., Fu R., Zhang Z. (2020). MicroTom Metabolic Network: Rewiring Tomato Metabolic Regulatory Network throughout the Growth Cycle. Mol. Plant.

[91] Chen T., Zhang Z., Li B., Qin G., Tian S. (2021). Molecular basis for optimizing sugar metabolism and transport during fruit development. aBIOTECH.

[92] You Y., van Kan J.A.L. (2017). Bitter and sweet make tomato hard to (b)eat. New Phytol..

[93] Fraser P.D., Bramley P.M. (2004). The biosynthesis and nutritional uses of carotenoids. Prog. Lipid Res..

[94] Zuluaga D.L., Gonzali S., Loreti E., Pucciariello C., Degl’Innocenti E., Guidi L., Alpi A., Perata P. (2008). Arabidopsis thaliana MYB75/PAP1 transcription factor induces anthocyanin production in transgenic tomato plants. Funct. Plant Biol..

[95] Yan S., Chen N., Huang Z., Li D., Zhi J., Yu B., Liu X., Cao B., Qiu Z. (2020). Anthocyanin Fruit encodes an R2R3-MYB transcription factor, SlAN2-like, activating the transcription of SlMYBATV to fine-tune anthocyanin content in tomato fruit. New Phytol..

[96] Jian W., Cao H., Yuan S., Liu Y., Lu J., Lu W., Li N., Wang J., Zou J., Tang N. (2019). SlMYB75, an MYB-type transcription factor, promotes anthocyanin accumulation and enhances volatile aroma production in tomato fruits. Hortic. Res..

[97] Butelli E., Titta L., Giorgio M., Mock H.-P., Matros A., Peterek S., Schijlen E.G.W.M., Hall R.D., Bovy A.G., Luo J. (2008). Enrichment of tomato fruit with health-promoting anthocyanins by expression of select transcription factors. Nat. Biotechnol..

[98] Mes P.J., Boches P., Myers J.R., Durst R. (2008). Characterization of Tomatoes Expressing Anthocyanin in the Fruit. J. Am. Soc. Hortic. Sci..

[99] Jones C.M., Mes P., Myers J.R. (2003). Characterization and Inheritance of the Anthocyanin fruit (Aft) Tomato. J. Hered..

[100] Tieman D., Zhu G., Resende M.F.R., Lin T., Nguyen C., Bies D., Rambla J.L., Beltran K.S.O., Taylor M., Zhang B. (2017). A chemical genetic roadmap to improved tomato flavor. Science.

[101] Alseekh S., Tong H., Scossa F., Brotman Y., Vigroux F., Tohge T., Ofner I., Zamir D., Nikoloski Z., Fernie A.R. (2017). Canalization of Tomato Fruit Metabolism. Plant Cell.

[102] Tohge T., Scossa F., Wendenburg R., Frasse P., Balbo I., Watanabe M., Alseekh S., Jadhav S.S., Delfin J.C., Lohse M. (2020). Exploiting Natural Variation in Tomato to Define Pathway Structure and Metabolic Regulation of Fruit Polyphenolics in the Lycopersicum Complex. Mol. Plant.

[103] Li J., Scarano A., Gonzalez N.M., D’Orso F., Yue Y., Nemeth K., Saalbach G., Hill L., Martins C.D.O., Moran R. (2022). Biofortified tomatoes provide a new route to vitamin D sufficiency. Nat. Plants.

[104] Food Data Central USDA. https://fdc.nal.usda.gov/.

[105] Ezura H. (2022). The world’s first CRISPR tomato launched to a Japanese market: The social-economic impact of its implementation on crop genome editing. Plant Cell Physiol..

[106] El-Mogy M.M., Atia M.A.M., Dhawi F., Fouad A.S., Bendary E.S.A., Khojah E., Samra B.N., Abdelgawad K.F., Ibrahim M.F.M., Abdeldaym E.A. (2022). Towards Better Grafting: SCoT and CDDP Analyses for Prediction of the Tomato Rootstocks Performance under Drought Stress. Agronomy.

